# At the bottom of the differential diagnosis list: unusual causes of pediatric hypertension

**DOI:** 10.1007/s00467-008-0744-y

**Published:** 2008-03-05

**Authors:** Matthew M. Grinsell, Victoria F. Norwood

**Affiliations:** Department of Pediatrics, Division of Nephrology, University of Virginia, 1224 Jefferson Park Ave., Charlottesville, VA 22903 USA

**Keywords:** Immobilization, Trauma, Abdominal wall defects, Drugs, Herbal therapies, Genetic disorders, Hypertension

## Abstract

Hypertension affects 1–5% of children and adolescents, and the incidence has been increasing in association with obesity. However, secondary causes of hypertension such as renal parenchymal diseases, congenital abnormalities and renovascular disorders still remain the leading cause of pediatric hypertension, particularly in children under 12 years old. Other less common causes of hypertension in children and adolescents, including immobilization, burns, illicit and prescription drugs, dietary supplements, genetic disorders, and tumors will be addressed in this review.

## Introduction

Despite a recent increase in the diagnosis of pediatric hypertension due to the proliferation of obesity, hypertension is relatively uncommon in the pediatric age group, affecting 1–5% of children [1]. In contrast to adults, hypertension in children is usually secondary to an identifiable etiology, with the most common cause being renal disease [2]. When evaluating a pediatric patient for hypertension, it is reasonable to first consider the common etiologies such as renal parenchymal diseases, congenital abnormalities, and renovascular disorders. However, a number of uncommon processes can also cause hypertension in the pediatric population. Each of these etiologies is rare in and of itself, and the number of patients with “unusual” causes will comprise less than 5% of all patients with secondary hypertension. This review focuses on select unusual etiologies (Table 1). Often, careful history and consideration of the context or the presentation of hypertension may be important clues to the diagnosis in these less frequent processes.

**Table 1:** Secondary causes of hypertension

Acute	Chronic
Renal	Renal
Acute poststreptococcal glomerulonephritis	Chronic nephritis or kidney disease, any cause
Hemolytic uremic syndrome	Obstructive uropathy
Acute nephritis, any cause	Polycystic kidney disease
Interstitial nephritis	Multicystic dysplastic kidney
Acute kidney injury	
Urinary tract obstruction	
Vascular	Vascular
Renal artery thrombosis	Renal artery stenosis
Patent ductus arteriosus	Aortic coarctation
Renal vein thrombosis	Vasculitis
	Williams syndrome
Ingestions	Ingestions
Prescription medications^a^	Prescription medications^a^
Over-the-counter medications	Over-the-counter medications
Illicit drugs^a^	Illicit drugs^a^
Herbal therapies^a^	Dietary supplements^a^
Surgical/trauma/injury	Endocrine/tumors
Immobilization/traction^a^	Pheochromocytoma^a^
Hypercalcemia^a^	Renal trauma^a^
Burns^a^	Neuroblastoma^a^
Renal trauma	Juxtaglomerular cell tumors^a^
Abdominal wall defect closure^a^	Mesoblastic nephroma
Orthopedic limb lengthening^a^	Hyper-/hypothyroidism
Intravascular volume overload	Congenital adrenal hyperplasia
	Mineralocorticoid excess
Neurologic	Genetic syndromes or inherited disorders
Pain	Williams syndrome^a^
Seizures	Tuberous sclerosis^a^
	Neurofibromatosis^a^
	Apparent mineralocorticoid excess
	Glucocorticoid remediable aldosteronism
	Liddle’s syndrome

^a^Etiologies covered in this review

## Immobilization

Hypertension is a widely reported complication of immobilization secondary to fractures, burns, or illness [3–8]. In some studies, over half of children in skeletal traction develop hypertension [3, 5, 7]. When this form of hypertension occurs, it has been reported to manifest from 1 to 11 weeks following onset of immobilization [3, 6, 9, 10]. Although hypertension may develop in any patient during prolonged immobilization, school-age and adolescent males appear to be particularly at risk [3, 6, 9, 11]. The pathophysiology of immobilization hypertension is unclear and likely multifactorial. Two possible mechanisms that have been investigated include hypercalcemia and increased sympathetic activity.

### Hypercalcemia

Immobilization due to fractures, burns, paralysis, or other causes is known to induce marked changes in calcium metabolism that may include hypercalcemia (>10.0 mg/dl), hypercalciuria (urinary calcium/creatinine ratio >0.3), elevated serum ionized calcium, and, if immobilization is prolonged, decreased total body calcium and loss of bone mineral density [4, 12–17]. It has been demonstrated that acute hypercalcemia during intravenous calcium infusion increases blood pressure and peripheral vascular resistance in both normotensive and hypertensive individuals [18, 19]. Whether or not alterations in calcium metabolism lead to immobilization-induced hypertension is less clear. Several case reports describe hypertension associated with marked hypercalcemia, although in some of these reports, comorbid conditions such as renal insufficiency may also have contributed [9, 20]. However, larger studies on hypertension in immobilized children have found no significant correlation between hypertension and hypercalcemia [3, 5]. Symptoms of hypercalcemia are nonspecific and include nausea, vomiting, constipation, seizures, and behavioral changes [11, 21]. If an immobilized patient develops hypertension, it is reasonable to assess serum total and ionized calcium, as ionized calcium may be elevated in immobilized patients even with normal total calcium levels [14].

The optimal treatment of immobilization-induced hypercalcemia has not been established. Increased fluid administration, loop diuretics, calcitonin, and bisphosphonates have all been shown to decrease serum calcium in the short term [16, 21, 22]. In the long run, however, increased activity with weightbearing exercise is most effective in inducing complete resolution of hypercalcemia and hypertension [4, 9, 10, 14, 16].

### Increased sympathetic activity

Patients in skeletal traction may develop hypertension relatively early in their hospital course, usually 1–4 days after immobilization [6, 23]. Increased sympathetic activity has been implicated as a cause based on elevated concentrations of circulating catecholamines and increased activity of the renin-angiotensin-aldosterone system (RAAS) [6]. One hypothesis has been that stretching of the sciatic nerve activates a vasomotor response leading to hypertension, and experimental stretching of the sciatic nerve in dogs increases blood pressure [23].

In general, hypertension associated with traction is mild to moderate and reversible, particularly with discontinuation or modification of traction [3, 5, 7, 24, 25]. Immobilized patients should have blood pressures monitored regularly. Volume status and the possibility of urinary retention should be assessed, especially if the injury involves the spinal cord. If traction modification is not feasible, drug therapy with α-blockers, dihydropyridine calcium-channel blockers, or direct vasodilators have been effective [5, 6, 20, 25]. Further evaluation for other causes of hypertension should be undertaken if blood pressure does not normalize within 7–10 days after discontinuation of immobilization.

## Trauma and burns

### Renal trauma

Posttraumatic hypertension can be separated temporally into acute presentations and chronic complications. In the acute setting, hypertension is a finding in up to 30% of patients with blunt renal trauma [26–29]. However, it may be difficult to determine whether the injured kidney itself is the source of hypertension or if other factors are involved for two closely related reasons. The first reason is that mild to severe blunt trauma in children from any cause significantly elevates systolic blood pressure approximately 20 mmHg above the 95 percentile for age [30]. The second reason is that the majority of children with renal injury suffer multiple traumas, which may contribute to hypertension due to pain and anxiety [26, 27, 29, 31, 32].

The incidence of posttraumatic hypertension outside the acute setting has been difficult to determine. Hypertension was reported as a complication following renal trauma in small series [27, 33]. However, larger, long-term follow-up studies have not found an increased incidence of hypertension in patients with a history of renal trauma compared with the general population [29, 34, 35]. Hutchison and Nogrady found no hypertension in 77 children aged 3–16 years who were followed for at least 2 years after renal trauma despite documented scarring in 20 of the 77 children [36]. Similarly, Surana et al. found no hypertension in 19 children followed for an average of 5.5 years after renal trauma [37].

When posttraumatic hypertension occurs, the mechanisms may include ischemia from arterial stenosis or occlusion; parenchymal compression due to perirenal hematoma, urinoma or scar; and traumatic arteriovenous fistulae [28]. Initial management of renal trauma is aimed at preserving renal function and minimizing patient morbidity. Recent reports demonstrated that nonoperative management of children with renal injury of any severity preserves renal function and is not associated with a higher complication rate [38–40].

Despite data showing that hypertension in children after renal trauma is uncommon, hypertensive patients with a history of renal trauma should undergo a focused evaluation. Damaged kidneys in young children with or without hypertension have been shown to demonstrate abnormal growth, so ultrasound evaluation of renal size is reasonable [36]. Other studies may include angiography to evaluate for vascular stenosis, cortical imaging to evaluate scarring, and renal vein renin assays. Therapy for posttraumatic hypertension should be aimed at the RAAS.

#### Burns

Hypertension has been reported within 7–10 days of hospitalization in up to half of children admitted for burns and is more common in patients with greater than 20% body surface area burns [41–43]. Popp et al. concluded that hypertensive burn patients were more likely to develop complications of encephalopathy and seizure compared with normotensive patients and required careful blood pressure monitoring [42].

The etiology of hypertension in burned children is not entirely clear but seems in part to be due to a complex neuroendocrine response. Increased activity of the RAAS has been observed in both hypertensive and normotensive pediatric burn patients and does not in itself account for hypertension [43]. Popp et al. found the main difference between hypertensive and normotensive burn patients to be volume status, with hypertensive patients having received overly aggressive fluid resuscitation [43]. Therefore, hypervolemia in a setting of active vasoconstriction from neurohumoral causes appears to be the most likely etiology of the hypertension [43].

Evaluation of burn patients with hypertension should focus on adequate analgesia and conscientious fluid management. Given the propensity of these patients to have hypertensive encephalopathy and seizures, aggressive treatment of high blood pressure is appropriate. Logical therapies for hypertension in these patients include but are not limited to diuretics, β-blockers, and angiotensin-converting enzyme inhibitors (ACE-i).

## Abdominal wall defects (AWD)

Surgical closure of AWD such as omphalocele or gastroschisis frequently results in hypertension and usually develops within 2 weeks after surgery [44, 45]. Hypertension occurs in nearly half of patients following closure of omphalocele compared with only 20% following closure of gastroschisis [44]. The etiology of the hypertension is unknown in these cases but is hypothesized to be due to increased intra-abdominal pressure leading to renovascular compression with ischemia and activation of the RAAS. Hypertension following closure of AWD is usually self-limited and lasts on average around 4 days for omphalocele patients and only 1 day for gastroschisis patients [44]. Hypertension following AWD closure is usually mild and self-limited, and its transient nature often makes treatment unnecessary. Approximately 10% of AWD patents may develop persistent hypertension requiring ongoing therapy. Whereas no specific recommendations exist for treatment, calcium-channel blockers, β-blockers, clonidine, and diuretics may be useful. If those medications are not effective, judicious use of ACE-i has been shown to be effective in neonatal hypertension. ACE-i use should be carefully monitored for alterations in urine output or renal function.

## Drugs

### Cocaine

Eight percent of high school seniors report using some form of cocaine in their lifetime, and cocaine use was implicated in 30% of all drug-related emergency room visits in 1999 [46, 47]. Following ingestion by intravenous, inhalation, or enteral routes, cocaine stimulates central and peripheral α-adrenergic receptors, inhibits catecholamine reuptake and nitric oxide synthesis, and subsequently results in marked vasoconstriction and hypertension [48–50]. Other physiologic effects of cocaine include increased heart rate and temperature, dilated pupils, and erratic behavior [51]. The duration of the effects of cocaine depend on the dose and route of administration but is generally less than 1 h. Therefore, outside of the emergency room setting, cocaine is a rare cause of hypertension.

Diagnosis of cocaine-induced hypertension is based primarily on clinical context, history, index of suspicion, and physical exam. A urine toxicology screen may be sent, but the results only indicate cocaine use within the last 3–5 days and may not be available in time to assist in making therapeutic decisions.

Therapy for cocaine toxicity should be aimed at reducing cardiovascular risks of myocardial infarction, angina pectoris, arrhythmias, and aortic dissection [48]. Alpha-blockers such as phentolamine and prazosin are recommended [47]. Other effective agents include sodium nitroprusside and nicardipine [48]. The use of β-blockers should be avoided because their use can lead to unopposed α-adrenergic activity and worsening of the hypertension [47, 48].

### Methamphetamine and 3,4 methylenedioxymethamphetamine (MDMA or ecstasy)

Around 5% of high school seniors admit to using methamphetamine and its synthetic cousin MDMA in their lifetimes [52]. These drugs are taken recreationally for their stimulant and hallucinogenic properties, with cardiovascular side effects of hypertension, increased heart rate, and elevated temperature. The major cause of morbidity and mortality related to methamphetamine use is intracerebral hemorrhage associated with malignant hypertension, hyperthermia, and coagulopathy [48, 52–54]. Methamphetamines produce their effects by increasing catecholamine release from presynaptic neurons and by weakly inhibiting inactivation of the catecholamines by monoamine oxidase [48]. In contrast to cocaine, methamphetamine is metabolized more slowly, resulting in a longer duration of action [48, 55].

Diagnosis and treatment of methamphetamine toxicity is similar to that of cocaine. Therapy should be aimed at safely reducing blood pressure. Dihydropyridine calcium-channel blockers, sodium nitroprusside, and α-blockers have all been recommended [48]. As with cocaine, β-blockers are discouraged because of the risk of unopposed alpha-adrenergic activity, leading to worsening of the hypertension.

### Prescription medications

Recreational use and abuse of prescription medications by adolescents is increasing, with approximately 10% of 12th graders reporting nonprescribed use of stimulants, opioids, and sedatives in 2005 [56, 57]. The prescription stimulants typically abused include dextroamphetamine (Dexedrine and Adderall) and methylphenidate (Ritalin and Concerta), which are prescribed for attention deficit/hyperactivity disorder, narcolepsy, and depression unresponsive to other medical therapies. These stimulants are chemically related to amphetamines and have similar physiologic effects, with hypertension, tachycardia, and vasoconstriction.

Diagnosing hypertension due to prescription drug abuse requires a high index of suspicion. A detailed history, including medications used by family members, may be of value. Therapy for hypertension due to prescription drug abuse is similar to that of the illicit stimulants above [56].

Hypertension due to toxicity of illicit or prescription drugs should be treated in the emergency room in conjunction with the assistance of experienced toxicology services.

## Herbal therapies

The use of herbal and complementary medicines is widespread among the pediatric population, with more than 20% of children with acute or chronic illness reporting the use of nonprescribed supplements [58, 59]. Herbal products are used for many reasons among children and adolescents, including therapy for acute and chronic illnesses, weight loss, improvement in energy and athletic performance, and because they are seen as more natural by caregivers [58, 60, 61]. A complete discussion of all herbal products with adverse cardiovascular effects is outside the scope of this article. However, two relatively common herbal supplements with well-described adverse effects on blood pressure are Ma Huang and licorice.

### Ma Huang (ephedra)

Ephedra contains the alkaloids ephedrine and pseudoephedrine and is sold under a multitude of trade names. Ephedra is used in the treatment of asthma, fever and chills, and headaches and is found in a large number of weight-loss formulations [60]. Ephedra is also used as a stimulant commonly known as “herbal ecstasy” [62]. Ephedra is usually consumed as a tea, although the formulation in weight-loss and dietary supplements is usually tablet or capsule form [60].

Following ingestion, ephedra is metabolized to norepinephrine, leading to a number of cardiovascular side effects including hypertension, tachycardia, arrhythmias, palpitations, myocardial infarction, and stroke [53, 60, 63, 64]. The diagnosis of ephedra toxicity may be difficult, as many patients and caregivers may not consider ephedra-containing products as medications and may not report its use.

Recommended therapy for acute ephedra toxicity is similar to that for cocaine and other sympathomimetics. Likewise, avoidance of β-blockers is prudent to prevent unopposed α-adrenergic activity and worsening of hypertension [53].

### Licorice

Licorice is an extract of the root of *Glycyrrhiza glabra* and is traditionally used for gastrointestinal complaints, bronchitis, coughs, and hepatitis [65]. Licorice may be ingested as a tea made form the plant root and may be found in candy, chewing tobacco, and gum. The active ingredient of licorice is glycyrrhizic acid which is subsequently metabolized to glycyrrhetinic acid, an inhibitor of 11β-hydroxysteroid dehydrogenase, the enzyme that normally catalyzes the inactivation of cortisol to cortisone [60, 66–68]. In chronic licorice ingestion, 11β-hydroxysteroid dehydrogenase inhibition leads to increased levels of cortisol, activation of renal mineralocorticoid receptors, and can lead to hypokalemia, sodium, and water retention, hypertension, and heart failure [60, 67]. In contrast to ephedra, hypertension with licorice generally requires chronic use.

Licorice-induced hypertension is reversible upon discontinuation of the compound, although inhibition of 11β-hydroxysteroid dehydrogenase and elevations in blood pressure may persist for up to 8 weeks [60, 66, 67, 69]. Hypertension from licorice responds to pharmacologic therapy with spironolactone and eplerenone in animal models [67, 70].

## Genetic syndromes

### Williams syndrome

Williams-Beuren syndrome, commonly referred to as Williams syndrome, is clinically characterized by “elfin facies”, mental retardation, infantile hypercalcemia, small stature, supravalvular aortic stenosis, and coarctation and stenosis of pulmonary and peripheral systemic arteries. Hypertension can therefore occur in the neonatal period associated with systemic hypercalcemia and later in childhood with the development of progressively worsening peripheral vascular stenoses and vessel wall compliance.

The responsible molecular defect is a large, multigenic deletion on the long arm of chromosome 7 (7q11.23) and includes the gene for elastin, the major protein component of the arterial wall [71]. Microscopic vascular pathology includes thickening of the arterial wall, disorganization of the elastic components, hypertrophy of smooth muscle cells, and abnormal orientation of collagen bundles [72].

Mutation of the elastin gene and subsequent vascular wall abnormalities are believed to be the cause of the vascular stenoses and therefore the cause of hypertension in up to 75% of patients. Recently, however, other genes located within the large deletion fragment have been associated with the development of hypertension. The *NCF1* gene encoding a subunit of nicotinamide adenosine dinucleotide phosphate oxidase is responsible for the generation of oxygen radicals and oxidative stress. When included in the mutated sequence of patients with Williams syndrome, the incidence of hypertension decreases, suggesting that oxidative stress exacerbates the development of hypertension in vessels with the molecular lesions of Williams syndrome [73].

Up to two thirds of children and adolescents with Williams syndrome will have hypertension due to renal artery stenosis, aortic coarctation, and thoracoabdominal aortic hypoplasia [73–75]. In most cases, the vascular lesions are multiple, and isolated renal artery stenosis in uncommon. Importantly, aortic stiffness is elevated and vessel compliance low in children with Williams syndrome, suggesting that intrinsic vascular abnormalities, probably due to the structural arterial wall abnormalities, contribute to the development of hypertension, even in the absence of stenotic lesions [75].

Treatment of hypertension in patients with Williams syndrome is difficult at best. Angioplasty is not recommended for stenotic lesions, as the majority of patients have multiple lesions, and there are no reports of long-term success with balloon procedures. The development of new lesions can be expected over time. ACE-i are likely to help control blood pressure in the presence of stenotic lesions but are associated with the risk of renal insufficiency when flow is compromised. Likewise, vasodilators have theoretical benefits, but the increasing knowledge of intrinsic vascular wall pathology and biochemical dysfunction perhaps explains the poor response to most pharmacologic treatments.

### Tuberous sclerosis (TSC)

TSC, an autosomal dominant process, is characterized by hamartomas of the brain, retina, heart, kidneys, and skin. Two genes may be mutated:* TSC1*, encoding hamartin, or* TSC2*, encoding tuberin [76]. Two thirds of cases appear to involve de novo mutations. In vivo, these two proteins form a heterodimer complex that controls growth, differentiation, and proliferation via inhibition of the target of rapamycin (mTOR) cascade [76]. Loss of functionality of either protein leads to activation of the proliferation cascade, but how this leads to the differentiation abnormalities of angiomyolipomas is unknown.

The usual renal lesion in TSC is multiple angiomyolipomas—benign tumors composed of abnormal vessels, immature smooth muscle cells, and fat cells—occurring in 55–75% of patients [76]. The prevalence of angiomyolipomas increases with age. Epithelial cysts can occur as single entities or as a part of the recently described TSC2/PKD1 contiguous gene syndrome, a more severe phenotype occurring in 2–3% of patients [77].

Angiomyolipomas are rarely the cause of hypertension but may occasionally result in compression of renal tissue or damage from hemorrhage. Hypertension is more commonly associated with epithelial cysts, especially in the more fulminant form of TSC2/PKD1 [76, 78]. Renovascular stenosis or aortic aneursyms occur rarely, and when seen, the lesions fit within the pathologic spectrum of fibromuscular dysplasia [79]. Of important note, all patients with TSC and hypertension should be evaluated for intracranial tubers, a common finding and a cause of hypertension when resulting in obstructive hydrocephalus.

The treatment of hypertension for patients with TSC is nonspecific unless an offending lesion can be surgically approached.

### Neurofibromatosis

Neurofibromatosis type 1 (NF1) is a common autosomal dominant disorder resulting from mesodermal and ectodermal dysplasia. The normal *NF1* gene product, neurofibromin, is a GTPase-activating protein that is ubiquitously expressed and regulates proliferation by inhibiting ras activity. Vascular lesions occur with an unknown frequency, as most are asymptomatic, and abnormalities include intimal proliferation, aneurysm formation, and arterial nodules. Renal arterial involvement is the most common site of symptomatic lesions and occurs in 1–2% of patients, although abnormalities may occur throughout the vascular tree [80]. Occasionally, compressive lesions from nearby fibromas may cause hypertension.

Hypertension is common in patients with NF1, and its prevalence increases with age. In children, the most commonly identified secondary cause is renal artery stenosis, whereas in adults, pheochromocytoma is more common [80]. Of note, the majority of hypertensive patients with NF1 do not have secondary hypertension, although the frequency of vasculopathy and its unknown pathophysiology make use of the term “essential” hypertension for these patients inappropriate. When actively investigated using ambulatory monitoring, the incidence of hypertension in young people with NF1 is approximately 16–19% [81, 82].

Given the incidence of renovascular lesions and the potentially catastrophic complications of pheochromocytoma, it is generally recommended that these abnormalities be ruled out in all hypertensive patients with NF1 [80]. For renal artery lesions, angiography is preferred over magnetic resonance imaging (MRI) due to its higher sensitivity in localizing small lesions, and renal vein renin sampling is recommended. Renal artery angioplasty for stenotic lesions is less successful in NF1 patients than in those without the disease but is a reasonable therapeutic strategy prior to more dramatic surgical options [80, 81, 83]. Given the high incidence of “essential” hypertension, it is possible that the lower success rates with angioplasty represent hypertension from more than one significant cause. The optimal pharmacologic management of hypertension in NF1 patients has not been determined, but multiple options seem effective.

## Tumors

### Neuroblastoma

Neuroblastomas are composed of neuroectodermal cells that arise from primitive sympathetic ganglion cells. During normal development, these cells migrate to become the adrenal medulla and parts of the sympathetic nervous system. These tumors are the most common extracranial solid neoplasms in childhood and are heterogenous, with highly variable clinical courses and histopathology. They most commonly arise in the adrenal gland but may also occur in abdominal, thoracic, cervical, or pelvic sympathetic ganglia. Hypertension may be a presenting feature of neuroblastoma and is due to excess catecholamine production and secretion by the mass. More commonly, mild hypertension may be noted as a part of the evaluation of a child with an abdominal mass, bone pain, or anemia [84]. The diagnosis is sealed with imaging, biopsy, and elevated urinary vanillylmandelic acid/homovanillic acid (VMA/HVA) levels without epinephrine/norepinephrine excess. Treatment with sympathetic blockers or clonidine is usually sufficient for hypertension management.

### Pheochromocytoma

Pheochromocytoma, derived from cells of neural crest origin, is the cause of hypertension in the pediatric age group in less than 1% of cases. Although most commonly sporadic, familial forms linked to Von-Hippel-Lindau (VHL), multiple endocrine neoplasia (MEN2), NF1, and succinate dehydrogenase B (SDHB) occur nearly twice as commonly in children (39%) than in adults [85]. Given the propensity for its association with inherited disorders, pheochromocytoma is thought to result from a “two-hit” phenomenon, especially in MEN2 and VHL. A recent study evaluated pheochromocytomas from 14 children, all of whom had significant chromosomal imbalances in tumor DNA. Ten of the patients carried constitutive mutations for VHL and SDHD [86].

Childhood pheochromocytoma is marked by sustained—or, rarely—paroxysmal hypertension and often presents with signs referable to central nervous system effects. Compared with adults, children have a higher frequency of bilateral or extra-adrenal tumors and a higher incidence of malignancy (12%) [85]. The diagnosis remains complex in pediatrics due to both the biochemical heterogeneity of the tumors and the scarcity of pediatric normal values for catecholamines and their derivatives, especially in the young child. Plasma and timed urine epinephrine, norepinephrine, VMA, metanephrine, and normetanephrine all have merit, with urine norepinephrine, normetanephrine, and VMA the most sensitive [85]. Plasma normetanephrine appears to be the most sensitive plasma assay [87]. Given the lack of standardized normal values for children, it should be noted that growth and age result in higher daily urine catecholamine excretions (expressed as μg/24 h). Therefore, normal adult values may be elevated for children [85]. Diagnosis through radiologic imaging is also likely to involve multiple modalities. Ultrasound is a reasonable screening tool, but MRI is utilized for more specific presurgical imaging. Given the likelihood of multiple lesions in children, ^131^I-metaiodobenzylguanidine (MIBG) imaging is also recommended to localize small or unusually placed tumors [85]. Treatment is classically described as sequential α-adrenergic/β-adrenergic blockade with phenoxybenzamine and propranolol, followed by surgical excision of the tumor. Labetalol has been an effective pharmaceutical agent in a number of cases, but it should be noted that labetalol interferes with the efficacy of MIBG scanning and can therefore complicate the evaluation [88]. Malignancy is difficult to predict based on macroscopic or microscopic morphology of resected tumors, resulting in the need for long-term follow-up for detection of metastases or new masses [88]. Similarly, development of future tumors is reasonably common, especially in the inherited disorders, and long-term follow-up is required.

### Juxtaglomerular cell tumors (JCT)

JCT are exceedingly rare, benign, renin-secreting tumors that develop from the smooth muscle cells of the afferent arterioles [89–93]. In addition to hypertension, patients with JCT also commonly present with signs of hyperaldosteronism, including hypokalemia, headache, polyuria, and vomiting [89–93]. JCT have a peak incidence in the second and third decades of life, with females affected slightly more often than males, although there have been cases in children as young as 5 years old [91, 92].

Diagnosis of JCT may require both laboratory and imaging modalities. One larger study found that renal vein renin did not lateralize in over half of the cases, although computed tomography demonstrated renal masses in all cases [92]. Surgery to remove all or part of the affected kidney cures hypertension associated with JCT [89, 92, 93]. Therapy with ACE-i or dihydropyridine calcium-channel blockers are effective in controlling blood pressure until surgery can be performed [91, 92].

## Summary

Evaluation of children and adolescents with hypertension should focus on determining the etiology of the hypertension and initiating appropriate therapy. It is important to note that patients with conditions associated with hypertension, such as immobilization, postoperative AWD, and burns, should be closely monitored for development of hypertension. In other settings such as the clinic or emergency room, careful history and physical exam, consideration of the context or presentation, and judicious use of laboratory and imaging studies may provide important clues to the diagnosis of rare causes of hypertension.

## Questions

(Answers appear following the reference list.)

### Immobilization

The association between hypertension and long-bone traction and immobilization is most likely due to:

- Intravascular volume expansion and sodium overload
- Pain
- Increased sympathetic activity and hypercalcemia
- Renal arterial compression and ischemia

### Trauma

The blood pressure pattern most commonly seen following blunt trauma is:

- Transient hypertension followed by normal blood pressure long term
- Normal blood pressure during the acute phase followed by permanent hypertension
- Severe hypertension acutely followed by slow resolution over years
- No clinically significant changes in blood pressure at any point

### Burns

Hypertension in burn patients should be aggressively treated because of an increased incidence of:

- Cardiac ischemia
- Encephalopathy and seizures
- Increased intracranial pressure and stroke
- Electrolyte disarray and arrhythmias

### Abdominal wall defects

Hypertension associated with an abdominal wall defect and its repair is most commonly:

- Mild and transient
- Severe and transient
- Mild and persistent
- Severe and persistent

### Stimulant medications

Which of the following is appropriately avoided in the treatment of stimulant-induced hypertension?

- Phentolamine
- Propranolol
- Nicardipine
- Sodium nitroprusside

### Genetic syndromes

The etiology of hypertension in William’s syndrome may be:

- Hypercalcemia
- Vascular stenoses
- Decreased vessel wall compliance
- All of the above

### Tumors

Accurate localization of pheochromocytomas usually requires imaging with both:

- MAG3 and MIBG
- DMSA and MRI
- MIBG and MRI
- CT and glucoheptonate
